# Identification and characterization of microRNAs involved in ascidian larval metamorphosis

**DOI:** 10.1186/s12864-018-4566-4

**Published:** 2018-03-01

**Authors:** Xiaoming Zhang, Xiaozhuo Liu, Chengzhang Liu, Jiankai Wei, Haiyan Yu, Bo Dong

**Affiliations:** 1 Ministry of Education Key Laboratory of Marine Genetics and Breeding, College of Marine Life Sciences, Ocean University of China, Qingdao, 266003 China; 2 Laboratory for Marine Biology and Biotechnology, Qingdao National Laboratory for Marine Science and Technology, Qingdao, 266237 China; 3 Key Laboratory of Experimental Marine Biology, Institute of Oceanology, Chinese Academy of Sciences, Qingdao, 266071 China; 4 Institute of Evolution and Marine Biodiversity, Ocean University of China, No. 5 Yushan Road, Qingdao, 266003 People’s Republic of China

**Keywords:** microRNAs, *Ciona savignyi*, Metamorphosis, Mesenchyme, Signaling pathways

## Abstract

**Background:**

Metamorphosis takes place within the life cycle of most marine invertebrates. The marine ascidian is a classical model to study complex cellular processes and underlying molecular mechanisms involved in its larval metamorphosis. The detailed molecular signaling pathways remain elusive, though extracellular signal-regulated kinases (ERKs) and c-Jun N-terminal kinase (JNK) have been revealed to regulate cell migration, differentiation, and apoptosis in ascidian larval organ regression and juvenile organ development. MicroRNAs (miRNAs) are small non-coding RNAs that modulate gene expression at the post-transcriptional level. Large numbers of miRNAs have been demonstrated to be involved in many developmental and metamorphic processes. However, the identification of miRNAs in ascidian larval metamorphosis has not yet been investigated.

**Results:**

Totally, 106 known and 59 novel miRNAs were screened out through RNA-sequencing of three small RNA libraries from 18 to 21-h post-fertilization (hpf) tailbud embryos as well as from 42 hpf larvae (after tail regression) in *Ciona savignyi*. Expression profiling of miRNAs was confirmed by quantitative real-time PCR, showing that the expression levels of csa-miR-4040, csa-miR-4086, csa-miR-4055, csa-miR-4060, csa-miR-216a, csa-miR-216b, csa-miR-217, csa-miR-183, and csa-miR-92c were significantly higher in 42 hpf larvae, whereas those of csa-miR-4018a, csa-miR-4018b, and csa-miR-4000f were higher in 18 and 21 hpf embryos; then, their expression in 42 hpf larvae became significantly low. For these 12 miRNAs, whose expression levels significantly changed, we predicted their target genes through the combination of miRanda and TargetScan. This prediction analysis revealed 332 miRNA-target gene pairs that were associated with the ERK, JNK, and transforming growth factor beta signaling pathways, suggesting that the identified miRNAs are involved in the regulation of *C. savignyi* larval metamorphosis via controlling the expression of their target genes. Furthermore, we validated the expression of five selected miRNAs by northern blotting. Among the selected miRNAs, the expression patterns of csa-miR-4018a, csa-miR-4018b, and csa-miR-4000f were further examined by whole-mount in situ hybridization. The results showed that all three miRNAs were specifically expressed in a cell population resembling mesenchymal cells at the head and trunk part in swimming larvae but not in metamorphic larvae. Utilizing the luciferase assay, we also confirmed that miR-4000f targeted *Mapk1*, suggesting that the csa-miR-4018a/csa-miR-4018b/csa-miR-4000f cluster regulates larval metamorphosis through the *Mapk1*-mediated signaling pathway.

**Conclusions:**

Totally, 165 miRNAs, including 59 novel ones, were identified from the embryos and larvae of *C. savignyi*. Twelve of them showed significant changes in expression before and during metamorphosis. In situ hybridization and northern blotting results revealed that three miRNAs are potentially involved in the signaling regulatory network for the migration and differentiation of mesenchymal cells in larval metamorphosis. Furthermore, the luciferase reporter assay revealed that *Mapk1* is a target of csa-miR-4000f. Our results not only present a list and profile of miRNAs involved in *Ciona* metamorphosis but also provide informative cues to further understand their function in ascidian larval metamorphosis.

**Electronic supplementary material:**

The online version of this article (10.1186/s12864-018-4566-4) contains supplementary material, which is available to authorized users.

## Background

Larval metamorphosis is a key stage in ascidian development. Complex cellular processes and tissue morphogenesis are involved in larval tail regression and new organ formation in juveniles. The larval trunk mesenchyme derived from the A7.6 (trunk lateral cells, TLCs), B8.5 and B7.7, as well as B7.5 (trunk ventral cells, TVCs) blastomeres of the 110-cell embryo is essential for making diverse adult organs including tunic cells, blood cells, muscles, and the heart [1]. The differentiation and migration of mesenchymal cells are highly coordinated spatially and temporally by a complex combination of signaling pathways, yet the underlying mechanisms remain unclear. The signaling pathways involved in *Ciona* larval tail regression initiation and propagation have been revealed in the past years. The epithelial growth factor (EGF) pathway plays an important role in early larval metamorphosis [2–4]. The mitogen-activated protein (MAP) kinases ERK and c-Jun N-terminal kinase (JNK) activate and regulate their downstream gene expression in tail tissues to drive the initiation of tail regression [5, 6]. Nitric oxide regulates tail regression through the JNK signaling pathway [7, 8]. However, little is known regarding molecular regulatory mechanisms on the origin and formation of juvenile tissues during metamorphosis.

miRNAs are a class of endogenous small non-coding RNA molecules that regulate the post-transcriptional expression of target genes by translational repression or transcript cleavage. They are involved in numerous biological processes, such as cell proliferation, apoptosis, growth, and migration [9–11]. Previous studies have shown that miRNAs are necessary for regulating metamorphosis in insects [12, 13]. Hundreds of miRNAs have been identified or predicted in tunicates, including *Oikopleura dioica* [14], *Ciona intestinalis* [15, 16], *Didemnum vexillum* [17], and *Halocynthia roretzi* [18]. Comparing miRNAs within most phyla, tunicates gained many novel non-conserved miRNA families [19]. Within the tunicate lineage, *Ciona* retained the major chordate miRNA families and most *C. intestinalis* miRNA families are conserved in *C. savignyi* [16]. Although numerous ascidian miRNAs have been identified, few miRNAs have been experimentally validated, and the biological functions of most miRNAs remain poorly understood. Thus far, miR-124 has been demonstrated to promote nervous system development by interacting with the Notch pathway in *C. intestinalis* embryogenesis [20]. However, little is known about the role of miRNAs in ascidian larval metamorphosis.

In the present study, three miRNA libraries from different stages of *C. savignyi* were constructed, and 106 known and 59 novel miRNAs were identified. The expression profiles of 78 miRNAs were validated, revealing that the expressional levels of 12 miRNAs showed significant differences before and during metamorphosis. In addition, the target genes of these 12 miRNAs were predicted. Furthermore, the expression patterns of three miRNAs were examined by in situ hybridization and, it was found that they were specifically expressed in trunk mesenchymal cells, indicating that they participated in new tissue formation in *Ciona* larval metamorphosis.

## Results

### Identification of miRNAs from three miRNA libraries of *C. savignyi* larvae

**Fig. 1:**
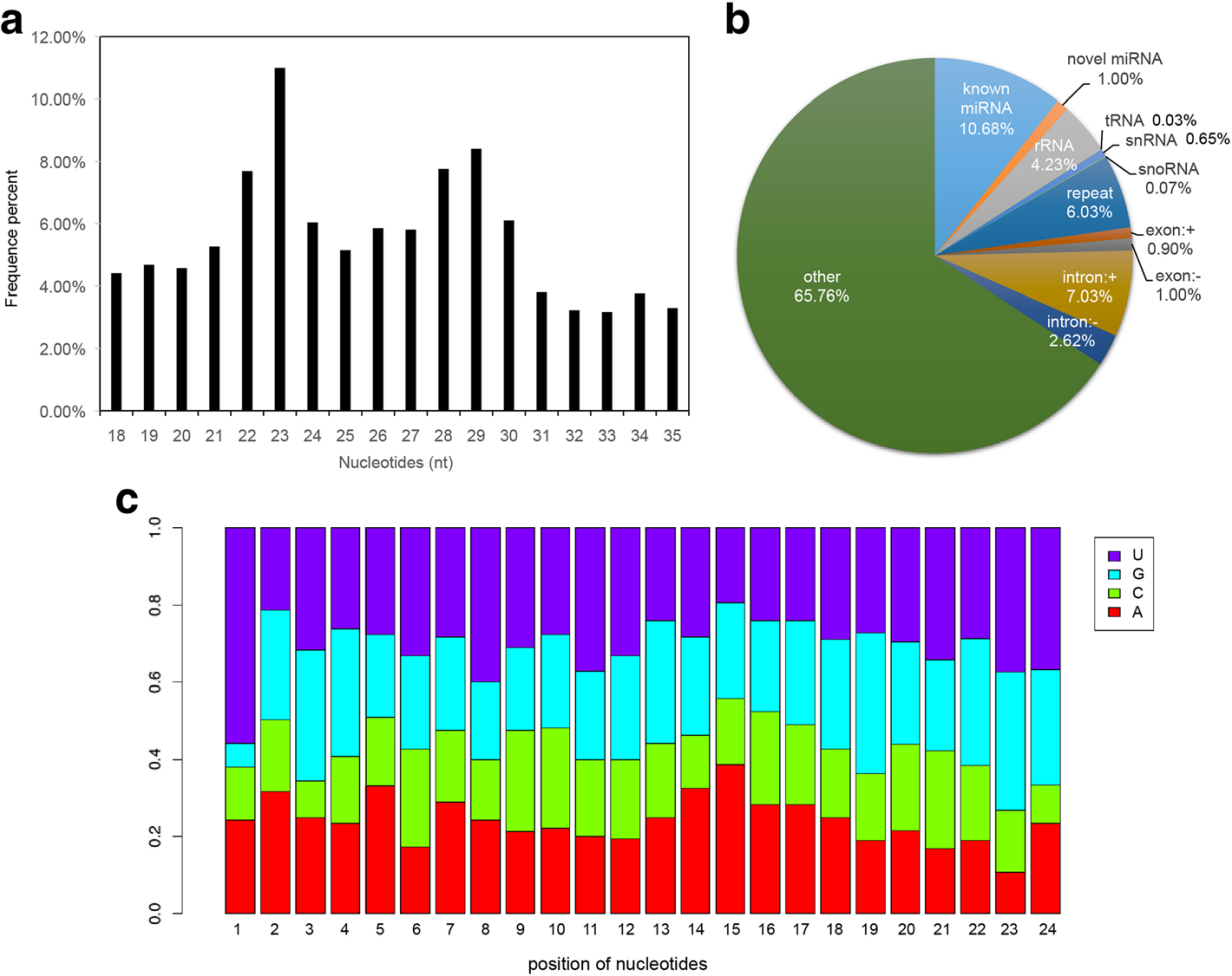
Characterization of sequenced data from three miRNA libraries of *Ciona savignyi* larvae. **a** Size distribution of sequenced small RNAs. **b** The classification and abundance of small RNA sequencing reads. Known miRNA, miRNAs that align to miRNAs in miRBase; novel miRNA, miRNAs that do not match any known miRNA/RNA sequence; rRNA, ribosomal RNA; tRNA, transfer RNA; snRNA, small nuclear RNA; snoRNA, small nucleolar RNA; repeat, repeat associate RNA; exon:+, exon in the DNA sense strand; exon:-, exon in the DNA antisense strand; intron:+, intron in the DNA sense strand; intron:-, intron in the DNA antisense strand; other, sequences do not match above classifications. **c** The frequency of miRNA nucleotide bases (A/U/C/G) at each position. Purple represents U, blue represents G, green represents C, and red represents A

To identify miRNAs that participated in *C. savignyi* metamorphosis, three miRNA libraries from larvae before (18 and 21 h post fertilization, hpf) and during (42 hpf) metamorphosis were constructed and sequenced. The total reads were 34,683,647. Nucleotide length distributions of sequenced small RNAs were analyzed by Solexa sequencing. Most sequenced small RNAs were 21–30 nucleotides in length (Fig. 1a). After comparing sequenced small RNA sequences with the existing *C. savignyi* mRNA database, 11,283,372 reads were mapped on the reference sequence. Then, 2,544,546 reads, which encode mRNA, rRNA, tRNA, snoRNA, repeats, introns, and exons, were removed (Fig. 1b). The remaining 8,738,826 reads were further analyzed through comparison with known miRNAs (miRNA precursors and mature miRNAs) in miRBase 13.0. A total of 106 known miRNAs (Additional file 1: Table S1), which were homologous to identified miRNAs from other species, and 59 novel ones (Additional file 2: Table S2) that did not match any known miRNA in miRBase were identified. Nucleotide base (A/U/C/G) analysis showed that the dominant first preferred nucleotide base at the first position was U (Fig. 1c).

### Origin and evolution of identified miRNAs

**Fig. 2:**
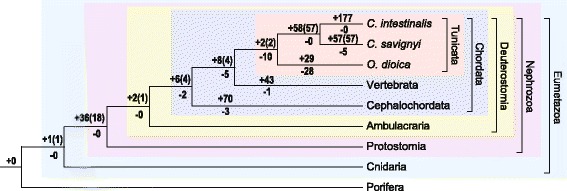
Evolutionary history of tunicate miRNAs. *C. savignyi* miRNAs were compared with miRNAs of 16 other animal species from miRBase. Only miRNAs supported by experimental evidence were used. An miRNA family was defined as acquired at the origin of a taxon if it is shared by at least two daughter lineages of the taxon but if is absent in all ancestor and sister lineages. At the base of each taxon, numbers are shown for the miRNA families acquired (+) and lost (−) during the origin of this taxon. A number is also given in parentheses to indicate the acquired families that are present in *C. savignyi* (not lost in evolution)

A total of 150 miRNA families were identified for *C. savignyi*, including 93 conserved and 57 novel families. To trace the evolutionary origin of these miRNAs, miRNA conservation across the phylogeny of metazoans was explored (Fig. 2). miR-10 was acquired at the base of Eumetazoa. Eighteen families evolved during the origin of Nephrozoa (miR-1, 7, 8/200, 25/92, 31, 34, 46/281, 96, 124, 125, 133, 153, 183, 184, 219, 281, 375, and let-7). One family was acquired at the base of Deuterostomia (miR-367). Four families evolved at the origin of Chordata (miR-135, 141, 217, 4057). The miR-1497 and 1502 families evolved at the origin of Tunicata, and 58 miRNA families were added at the base of *Ciona*.

### Expression profiles of the identified miRNAs

**Fig. 3:**
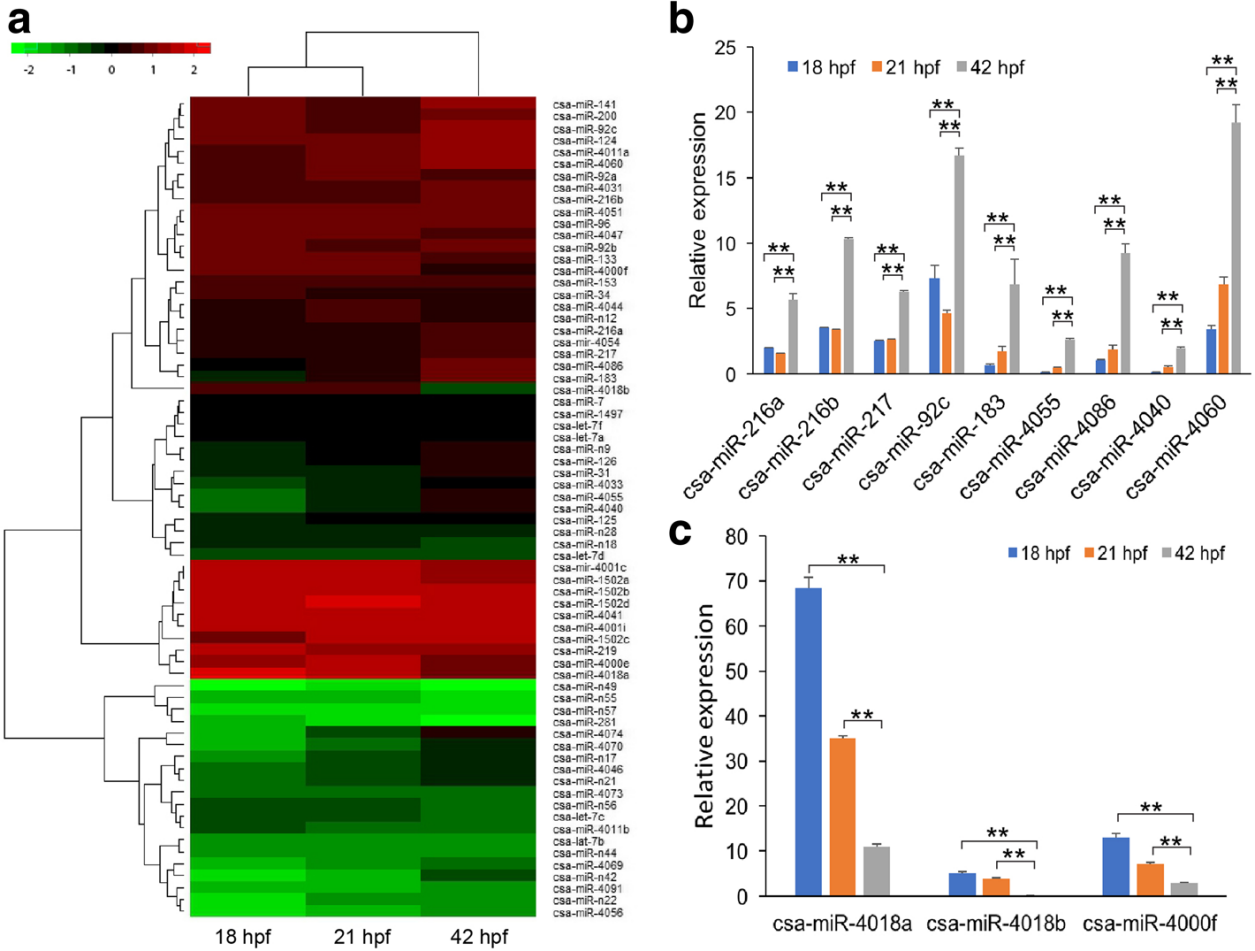
miRNA expression profiles before and after *C. savignyi* larval metamorphosis. **a** Heat map of relative expression levels of miRNA of *C. savignyi* larvae at 18, 21, and 42 hpf stages detected by qPCR. Each row represents an miRNA, and each column represents a specific stage indicated at the bottom. The expression of miRNA was hierarchically clustered on the left, and the sample clustering was presented at the top. The values shown are averages of independent experiments performed in triplicate. The color bar depicts the color contrast level of the heat map. Green or red on the heat map indicates a decrease or increase in miRNA levels, respectively. **b**-**c** The expression levels of upregulated miRNAs (**b**) and downregulated miRNAs (**c**) at 42 hpf were identified by qPCR. The relative expression of each miRNA was calculated using the delta-delta Ct method (2^−ΔΔCt^) and normalized to U6 snRNA. Results represented the mean of triplicate assays with standard error (mean ± SD). Significance of relative expression at different stages was determined using Student’s *t*-test. Asterisks (**) represented statistical significance at *P* < 0.01. Blue, orange and gray bars represent expression levels at 18, 21, and 42 hpf of each miRNA, respectively

To screen those miRNAs that potentially participated in *C. savignyi* larval metamorphosis, the expression profiles of the identified miRNAs at different stages were validated using quantitative real-time PCR (qPCR). The heat map showed the expressional level of the identified miRNAs (Fig. 3a). The expression of miRNAs from 18 and 21 hpf embryos did not generally show any significant difference. However, for some miRNAs from 42 hpf larvae, their expression was significantly different compared with that at the earlier stages. Further characterized results showed that the expression levels of nine miRNAs (csa-miR-4040, csa-miR-4086, csa-miR-4055, csa-miR-4060, csa-miR-216a, csa-miR-216b, csa-miR-217, csa-miR-183, and csa-miR-92c) significantly increased at 42 hpf compared with those at 18 and 21 hpf (*P* < 0.01) (Fig. 3b). Conversely, the expression levels of three miRNAs (csa-miR-4018a, csa-miR-4018b, and csa-miR-4000f) significantly decreased in metamorphic larvae (*P* < 0.01) (Fig. 3c), suggesting that they play vital roles in *C. savignyi* larval metamorphosis.

### Interaction between the identified miRNAs and their target gene predictions

**Fig. 4:**
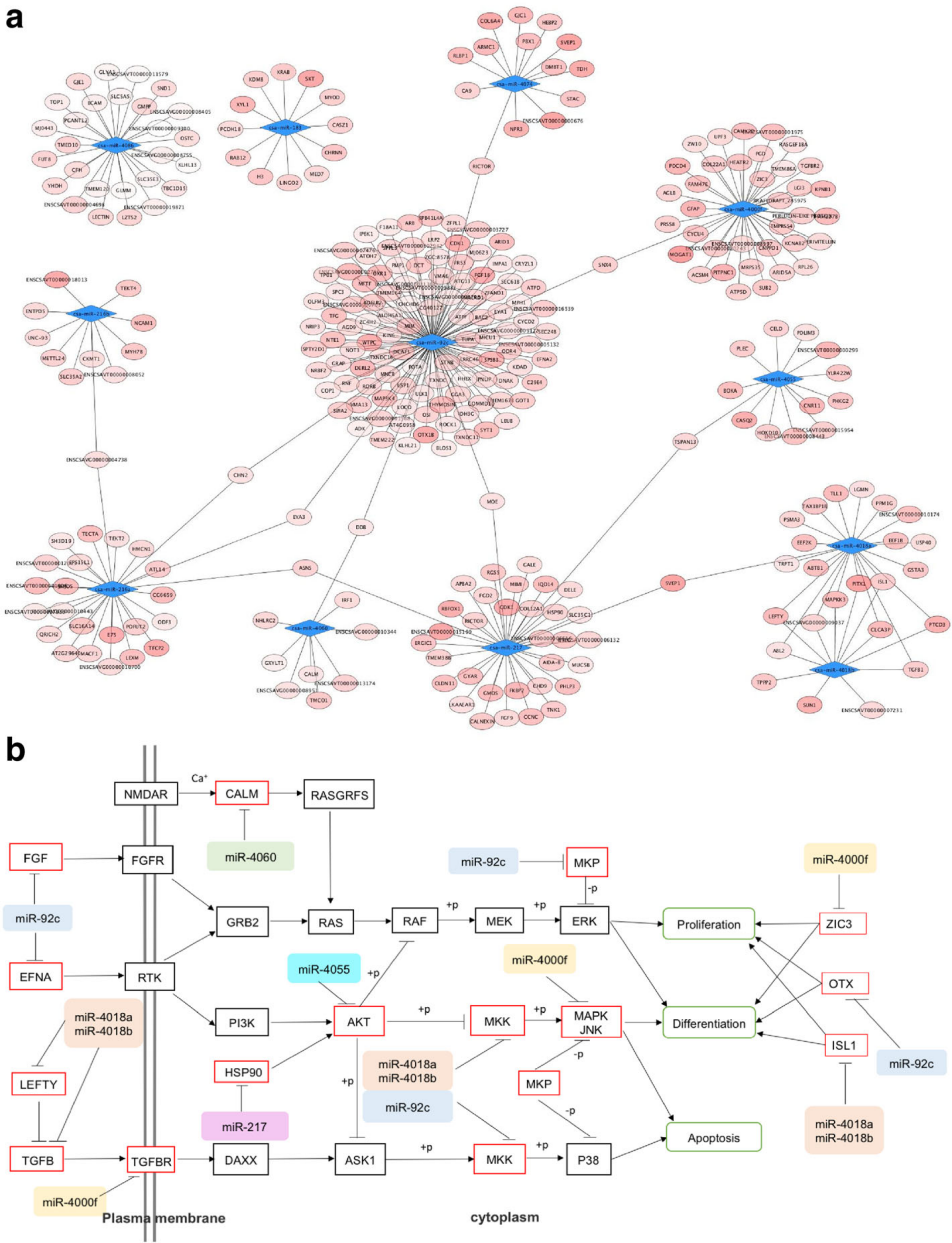
The interactions of identified miRNAs and their target gene prediction. **a** The interactions of 12 miRNAs and their target genes were visualized as a network using Cytoscape3.4. The target genes displayed in the network included the overlapping ones predicted by miRanda and TargetScan and those genes whose expression levels on performing RNA-Seq were negatively correlated with the expression of miRNAs. Nodes of blue diamonds and pink ovals represented miRNAs and their target genes, respectively. Gradient pink color in the nodes of the target reflects different free energy levels of the miRNA/target duplex (from dark pink to light pink for lower energy to higher energy). Black solid lines between nodes indicated the interrelationships of miRNAs. **b** Signal pathways related to *C. savignyi* metamorphosis targeted by miRNAs. Red line rectangles represent target genes; black line rectangles represent genes in a pathway; colored filled rectangles represent different miRNA; green line rounded rectangles represent biological processes; black arrows represent positive regulation; black line-bar represents negative regulation

For 12 miRNAs with either significantly higher or lower expression during larval metamorphosis, their target genes were predicted utilizing miRanda and TargetScan. Candidate target genes were from RNA-Seq data obtained from 18, 21, and 42 hpf embryos/larvae and the ENSEMBL database. Those genes overlapping in the prediction by the two softwares or their expressions in RNA-Seq negatively correlated with the 1 of the 12 miRNAs were picked as target genes. A total of 332 miRNA-target gene pairs were found and displayed by Cytoscape 3 in Fig. 4a. The results showed a complex network consisting of differentially expressed miRNAs and their target genes. Among them, csa-miR-92c targeted 114 genes including mitogen-activated protein kinase kinase (MKK) and mitogen-activated protein kinase phosphatase (MKP), which belong to the MAPK signaling pathway. Transforming growth factor (TGF) beta 1 (TGF-β1), sorting nexin 4, pituitary homeobox, and others were targeted by two miRNAs, and the target genes of miR-4018a and miR-4018b significantly overlapped.

Furthermore, KEGG pathway enrichment analysis was performed. The results showed that the MAPK, Ras-related protein 1 (Rap1), Ras, and TGF-β signaling pathways were involved in the target gene database (Fig. 4b). In the MAPK signaling pathway, csa-miR-4018a and csa-miR-4018b targeted MKK and csa-miR-92c targeted MKK and MKP. In the TGF-β signaling pathway, csa-miR-4018a and csa-miR-4018b commonly targeted the left-right determination factor and miR-4000f targeted the TGF-β receptor. In addition, miR-92c targeted FGF and Ephrin-A, which are upstream of the Rap1 and Ras signaling pathway.

It is worth noting that zinc finger protein of the cerebellum 3, orthodenticle homeobox, and insulin gene enhancer protein, which regulate pluripotency in stem cells [21–23], were targeted by csa-miR-4000f, csa-miR-92c, and csa-miR-4108a/csa-miR-4108b, respectively, revealing that they might control larval stem cell differentiation during metamorphosis. In addition, genes related to lysosome, endoplasmic reticulum, phagosome, endocytosis, and autophagy regulation were significantly enriched in KEGG results (Table 1), indicating that vesicle trafficking processes are involved in larval metamorphosis.

**Table 1:** KEGG pathway analysis of transport and catabolism

Pathway	Target Gene	KO ID	cDNA ID	miRNA
Endoplasmic	STT3	K07151	ENSCSAVG00000004164	csa-miR-4000f
Reticulum	CALR	K08057	ENSCSAVG00000000885	csa-miR-217
	MBTPS1	K08653	ENSCSAVG00000005178	csa-miR-4000f
	HYOU1	K09486	ENSCSAVG00000003312	csa-miR-92c
	HSP90B	K09487	ENSCSAVG00000010760	csa-miR-217
	PDIA6	K09584	ENSCSAVG00000010980	csa-miR-92c
	EDEM1	K10084	ENSCSAVG00000000652	csa-miR-92c
	SEC23	K14006	ENSCSAVG00000011363	csa-miR-92c
Phagosome	ATPeV1H	K02144	ENSCSAVG00000000896	csa-miR-92c
	ATPeV1A	K02145	ENSCSAVG00000005752	csa-miR-92c
	ATPeV0D	K02146	ENSCSAVG00000001895	csa-miR-92c
	ATPeV1F	K02151	ENSCSAVG00000011191	csa-miR-92c
	CALR	K08057	ENSCSAVG00000000885	csa-miR-217
	SEC61B	K09481	ENSCSAVG00000002561	csa-miR-92c
Endocytosis	SH3G	K11247	ENSCSAVG00000002074	csa-miR-216a
	ARFGAP2/3	K12493	ENSCSAVG00000001512	csa-miR-92c
	CCDC53	K18463	ENSCSAVG00000005922	csa-miR-92c
	VPS29	K18467	ENSCSAVG00000005941	csa-miR-92c
Lysosome	GALC	K01202	ENSCSAVG00000002286	csa-miR-92c
	ATPeV1H	K02144	ENSCSAVG00000000896	csa-miR-92c
	ATPeV0D	K02146	ENSCSAVG00000001895	csa-miR-92c
	AP1M	K12393	ENSCSAVG00000004175	csa-miR-92c
	LGMN	K01369	ENSCSAVG00000006624	csa-miR-4018a
	GGA	K12404	ENSCSAVG00000004489	csa-miR-92c
Autophagy	ULK1/2/3	K08269	ENSCSAVG00000003676	csa-miR-92c
	ATG13	K08331	ENSCSAVG00000003971	csa-miR-92c

### Validation of the identified miRNA expression levels

**Fig. 5:**
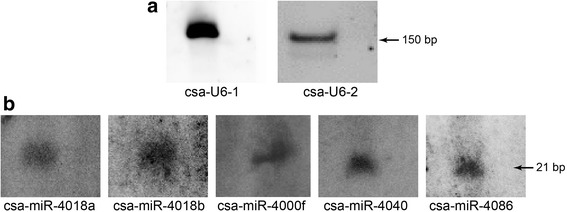
Validation of the identified miRNA existence and their expression by northern blotting. **a** U6 snRNA served as the loading control. Csa-U6–1 and csa-U6–2 using total RNA extracted from embryos and adult total RNA, respectively. **b** Northern blot analysis of csa-miR-4018a, csa-miR-4018b, csa-miR-4040, csa-miR-4086, and csa-miR-4000f was performed in denaturing gels using LNA probes. The size of mature miRNAs was approximately 21 nucleotides

The above screened 12 miRNAs included seven miRNAs which were annotated from bioinformatics analysis [16], but were not previously verified by experiments. To further validate the existence and expression of those identified miRNAs, northern blotting of seven miRNAs was conducted using RNA extracted from *C. savignyi*. Locked nucleic acid (LNA) probes were designed to detect miRNAs. U6 snoRNA was used as the loading control (Fig. 5a). Five probes from csa-miR-4040, csa-miR-4086, csa-miR-4000f, csa-miR-4018a, and csa-miR-4018b showed bands. The size of each band was consistent with that of mature miRNAs (21–24 nucleotides in length) (Fig. 5b and Additional file 3: Figure S1), indicating that these five miRNAs exist in the *C. savignyi* genome and that the mature ones are expressed during larval metamorphosis.

### Expression patterns of miRNAs downregulated during metamorphosis

**Fig. 6:**
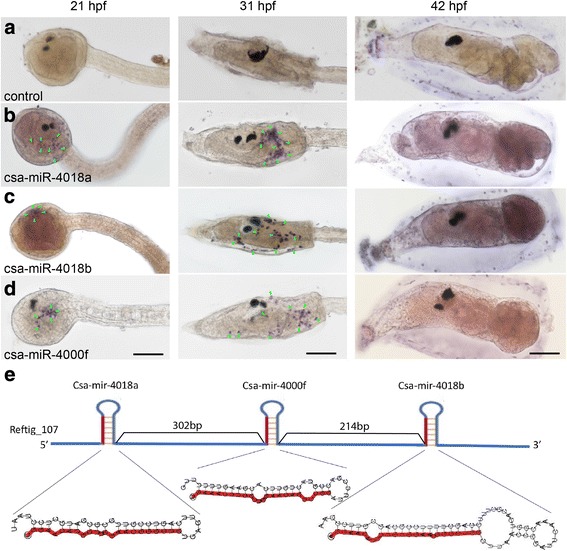
Expression patterns of three miRNAs detected by whole-mount in situ hybridization. Embryos and larvae at 21, 31, and 42 hpf were hybridized with LNA probes of scrambled control (**a**), csa-miR-4018a (**b**), csa-miR-4018b (**c**), and csa-miR-4000f (**d**). The developmental stages are indicated in column headings. Hybridized signals are indicated by green arrowheads. The expression signals of csa-miR-4018a, csa-miR-4018b, and csa-miR-4000f were specifically detected in a scattered cell population at 21 and 31 hpf, and their expression signals were stronger at 31 hpf but weaker at 42 hpf. (**e**). Schematic representation of the genomic localization of the precursors of csa-miR-4018a, csa-miR-4018b, and csa-miR-4000f. The numbers on the blue line represent the distance between two pre-miRNAs. Scale bars represent 100 μm

For miRNAs confirmed by northern blotting, their expression pattern was further examined in embryos and larvae utilizing whole-mount in situ hybridization. At 21 hpf, the expression of csa-miR-4018a, csa-miR-4018b, and csa-miR-4000f was detected. The results showed that they were all distributed in a scattered cell population at the head and trunk part in tailbud embryos (Fig. 6). At a later stage (31 hpf), stronger expression signals of all three miRNAs moved to the trunk part. During larval metamorphosis (42 hpf), a weak signal could be examined for the csa-miR-4018a probe (Fig. 6b). There were no detectable signals for the csa-miR-4018b and csa-miR-4000f probes (Fig. 6c-d). The expression patterns of the three miRNAs were similar and resembled that of mesenchymal cells [1]. Through a search of the genome scaffold, csa-miR-4018a, csa-miR-4018b, and csa-miR-4000f formed an miRNA cluster, consecutively located in scaffold reftig_107 (Fig. 6e), indicating that csa-miR-4018a, csa-miR-4018b, and csa-miR-4000f share similar regulatory elements. Significant expression changes of the three miRNAs in mesenchymal cells suggest that they play important roles in larval metamorphosis.

### Identification of Mapk1 as the target gene of miR-4000f

**Fig. 7:**
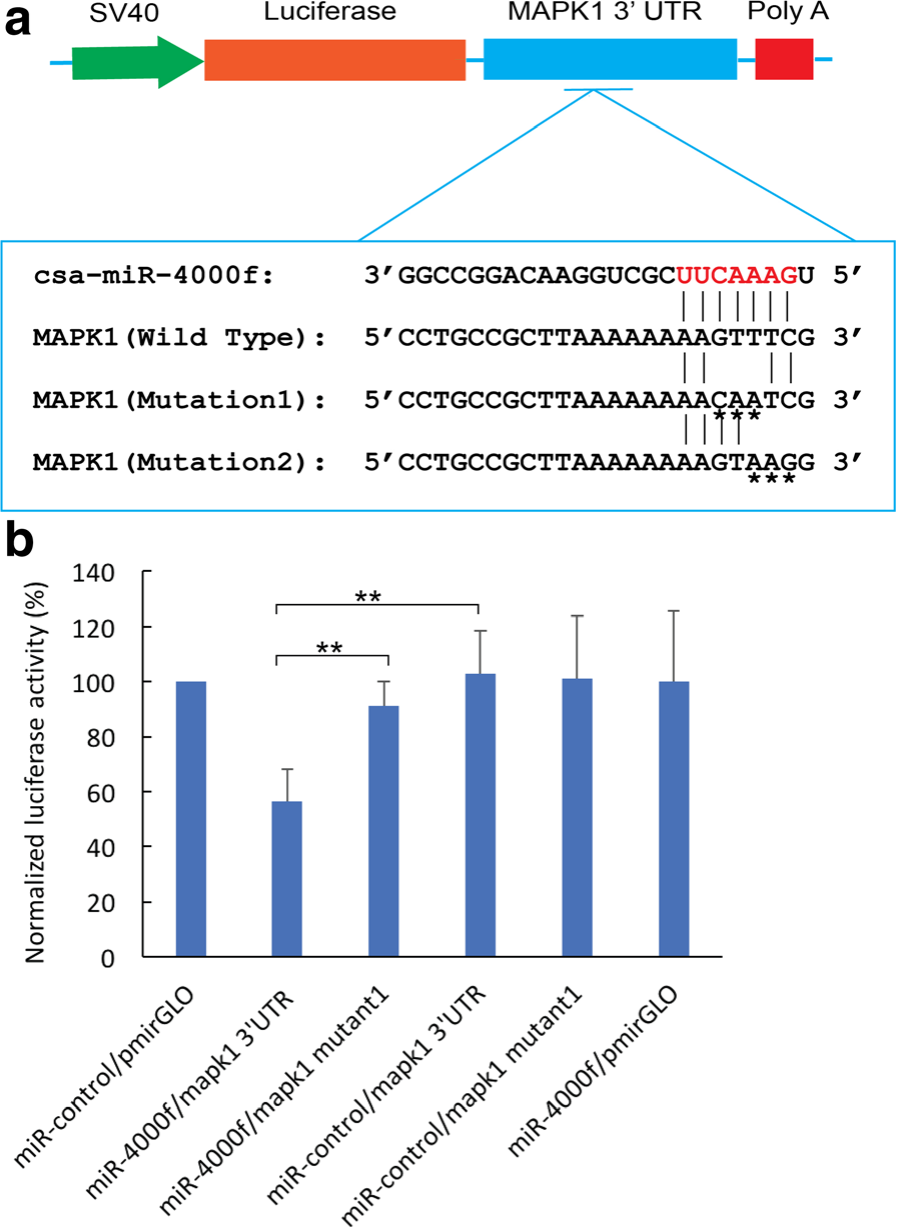
Csa-miR-4000f targets the *Mapk1* by interacting with its 3′-UTR. **a** Schema of luciferase reporter constructs carrying the 3′-UTR regions of target genes used in luciferase assays. *Mapk1–*3′-UTR or *Mapk1–*3′-UTR mutants were cloned into the downstream luciferase open reading frame of the pmirGLO dual luciferase vector. Sequence alignments of csa-miR-4000f and its predicted target gene *Mapk1–*3′-UTR or mutants of the *Mapk1–*3′-UTR are displayed in the blue line box. Asterisks (*) under letters indicate the mutant nucleotide in the *Mapk1–*3′-UTR mutant constructs. **b** Relative luciferase activity in HEK-293T cells co-transfected with pmirGLO-*Mapk1*–3′-UTR or pmirGLO-*Mapk1*–3′-UTR mutants or the empty vector pmirGLO and either csa-miR-4000f mimics or miR-control. Firefly luciferase values were normalized for transfection with *Renilla* luciferase activity. Student’s *t*-test was used to evaluate the significance of luciferase data. Asterisks (**) represented statistical significance at *P* < 0.01

miRNA target prediction suggested that both csa-miR-4018a and csa-miR-4018b targeted *Mapkk3* (Additional file 4: Figure S2A) and that csa-miR-4000f targeted *Mapk1* (Fig. 7a) through seed sequences. To investigate how the csa-miR-4018a/csa-miR-4018b/csa-miR-4000f cluster functions in larval metamorphosis, above prediction result was further experimentally confirmed through the luciferase assay. miRNA mimics or the miR-control was co-transfected into HEK293T cells with either its target gene 3′-UTR or 3′-UTR mutants, respectively. Luciferase activities were then measured at 48 h after each transfection. The results showed that normalized luciferase activities in cells co-transfected with csa-miR-4000f and pmirGLO-*Mapk1*–3′-UTR decreased 42% compared with that in miR-control and pmirGLO-*Mapk1*–3′- UTR co-transfected cells, whereas the relative luciferase activities in csa-miR-4000f and pmirGLO-*Mapk1*–3′-UTR mutant co-transfected cells did not significantly decrease (Fig. 7b), indicating that *Mapk1* is the target gene of csa-miR-4000f.

We also examined binding activities between csa-miR-4018a or csa-miR-4018b and *Mapkk3* utilizing the same approach. No significant reduction in luciferase activity appeared in csa-miR-4018a or csa-miR-4018b and pmirGLO-*Mapkk3*–3′-UTR co-transfected cells (Additional file 4: Figure S2B). These results suggest both csa-miR-4018a and csa-miR-4018b do not target *Mapkk3*.

## Discussion

In this study, 106 conserved miRNAs and 59 novel miRNAs were identified through the screening of three small RNA libraries from *C. savignyi* larvae before and during metamorphosis. The number of identified miRNAs was less than the number found in a previous study [16]. The reason was partly due to the utilization of embryos/larvae at three stages (18, 21, and 42 hpf) to construct libraries in our experiments. It has been found that lower levels of miRNA are expressed in late embryos and adults than in unfertilized eggs and early embryos [16].

Although species belonging to Tunicata have retained most deuterostome miRNAs, there were a significant number of miRNA families (miR-4, 19, 22, 50/190, 129, 137, 193, 210, 315, 365) that were lost at the base of the tunicate lineage compared with the only two families (miR-1497 and 1502) acquired during evolution. This is a rare phenomenon considering that miRNAs are seldom lost during evolution [24]. Moreover, another 28 conserved miRNAs seemed to be lost in the free-swimming tunicate *O. dioica.* The miRNA families of *Ciona* and *O. dioica* were surprisingly different as they shared only two tunicate-acquired miRNA families, while 61 and 31 new miRNAs originated in each branch, respectively, during the diversification of the two groups. As the evolution of miRNA is believed to correlate with morphological complexity [25], the dynamic miRNA repertoire in the tunicate lineage might be involved in the degeneration and rearrangement of body plans during metamorphosis. Expression profiling of miRNAs using qPCR showed that the expression of the three miRNAs acquired at the origin of Chordata (miR-141, 217) and the vertebrata plus tunicata (miR-126) lineages were all significantly upregulated at 42 hpf larvae, whereas the two Tunicata-acquired miRNAs (miR-1497, 1502) did not show such a pattern.

Among 165 identified miRNAs, 78 were further investigated by qPCR to confirm their temporal expression profile before and during *C. savignyi* larval metamorphosis. Twelve miRNAs were validated to be significantly either up- or downregulated at 42 hpf, indicating that they were involved in the regulation of larval metamorphosis. Upregulated miRNAs might function to inhibit their targets, such as MKP, which could hinder the progression of developmental events such as cell apoptosis. Metamorphosis involves numerous rapid morphogenetic movements and physiological changes and is regulated by several complicated signal pathways and gene networks. Thus, the lower expression of some miRNAs during metamorphosis could trigger their target genes, such as MKK, and result in obvious alterations in subsequent larval survival and adult tissue development.

Previously identified *Ciona* miRNAs were involved in unfertilized egg, embryo, and adult development [16]. In the present study, to the best of our knowledge, we have, for the first time, identified miRNAs that have potentially regulated larval metamorphosis. To uncover the biological roles of miRNAs in metamorphosis, we predicted their target genes and performed KEGG pathway enrichment analysis. A total of 332 miRNA-target gene pairs were predicted by miRanda and TargetScan. Some target genes, such as EGF and Sushi, have been reported to be important molecules during larval metamorphosis [4, 5]. In the present study, the MAPK, Rap1, Ras, EGF, and TGF-β signaling pathways were identified to be involved in the target gene database. Some elements of these pathways have been found during *Ciona* larval metamorphosis [6, 26], suggesting that miRNAs interact with their target genes to regulate tissue rearrangement during larval metamorphosis. In addition, **s**everal pathways relevant to vesicle formation, transportation, and secretion were identified. Recently, exosomes have been widely reported to mediate cell-to-cell communication. miRNAs are one of the important cargos of exosomes that play crucial roles in diverse developmental events [27, 28]. Thus, our data revealed that miRNAs might function in larval metamorphosis via extracellular exosome trafficking.

The cell population in which csa-miR-4018a, csa-miR-4018b, and csa-miR-4000f were expressed resembled mesenchymal cells in the *Ciona* larval trunk [1], which is essential for the formation of the heart, muscles, blood, and juvenile organs in metamorphic larvae. Ascidian larvae contain three lines of mesenchymal cells TLCs, TVCs, and mesenchyme [1]. At 31 hpf, csa-miR-4018a, csa-miR-4018b, and csa-miR-4000f showed different expression patterns. This could be caused either by the migration of mesenchymal cells in the trunk during larval development or by the expression of these three miRNAs in different subpopulations of the mesenchyme in ascidian larvae. In mammals, mesenchymal stem cells (MSCs) are multipotent stromal cells that differentiate into various cell types [29]. *Ciona* miRNAs expressed in mesenchymal cells might regulate new tissue development by interacting with their target genes. KEGG pathway enrichment analyses showed that their target genes were included in the genes of the MAPK, Rap1, Ras, and TGF-β signal pathways. We further confirmed that *Mapk1* is the target of csa-miR-4000f. During early embryogenesis in ascidians, MAPK-mediated FGF signaling is required to induce mesenchyme cell fate through the activation of different genes [30–32]. In *Ciona* larval metamorphosis, the MAPK pathway has been demonstrated to be required for tail regression [5, 6]. Our current results suggested that the csa-miR-4018a/csa-miR-4018b/csa-miR-4000f cluster regulates larval metamorphosis through the MAPK1-mediated signaling pathway.

Recent studies have implicated that exosomes are the key effectors of MSC function; exosomes are able to carry miRNAs and are released by MSCs to facilitate diverse functions [29, 33, 34]. Thus, we hypothesized that *Ciona* mesenchymal cells regulate adult tissue proliferation and differentiation by transferring miRNAs to recipient cells through the release of exosomes. Csa-miR-4018a, csa-miR-4018b, and csa-miR-4000f were located adjacent to each other on the same chromosome and formed a miRNA cluster. Generally, high sequence homology between the miRNAs in a cluster permit both common and unique mRNA targets. These miRNA targets lie within the same pathway, thereby allowing these miRNAs to regulate several components of a cellular process [35]. The similar expression patterns of these three miRNAs (Fig. 6) indicated that the members of this miRNA cluster were transcribed as a single precursor. It is interesting to further investigate how these three miRNAs contribute to larval metamorphosis by regulating cell differentiation and migration in the near future.

## Methods

### Animals, embryos, and larvae

*C. savignyi* adults were collected from the coast of Qingdao, China. The animals were kept and acclimated in aerated seawater for three days under constant light to accumulate gametes. Eggs were then dissected and mixed with seawater with sperm from other individuals. Five minutes after fertilization, eggs were washed with seawater through a nylon filter to remove sperm and debris. Embryos were cultured at 16 °C.

### Small-RNA library construction and sequencing

Ascidian embryos and larvae were collected at 18, 21, and 42 hpf and were then immediately frozen in liquid nitrogen. Total RNA was extracted with RNAiso Reagent (Takara). Totally, 3 μg of total RNA per sample was used as input material for the construction of small RNA libraries. Libraries were generated using NEBNext® Multiplex Small RNA Library Prep Set for Illumina® (NEB) following the manufacturer’s instructions and were sequenced on an Illumina Hiseq 2500/2000 platform at Novogene, China.

### qPCR

Reverse transcription was carried out using the Mir-X™ miRNA First-Strand Synthesis Kit (Clontech). QPCR amplification was performed using the Mir-X miRNA qPCR SYBR Kit (Clontech) on LightCycler 480 (Roche). The reaction conditions were as follows: 95 °C for 10 min, 40 cycles of 95 °C for 15 s, and 60 °C for 1 min. U6 snRNA served as an internal normalization control. The entire sequence of mature miRNA was used as a miRNA-specific 5′ primer. Data were analyzed using the comparative Ct method, and statistical analyses were performed using paired Student’s *t*-tests.

### Northern blot

Total RNA (8–20 μg) from 21 hpf embryos and adults were isolated using RNAiso Reagent (Takara) and electrophoresed in a denaturing 12% polyacrylamide gel and transferred onto a Hybond-N+ membrane (GE Healthcare). miRNA probes (Additional file 5: Table S3) were DIG-labeled LNA-modified probes synthesized by Exiqon (Denmark). Hybridization and washing were performed as described by Válóczi [36]. Signals were detected using CDP-*Star* chemiluminescent substrate (Roche), and the blot was exposed to the Lumi-Imager F1 Workstation. To control equal loading, blots were hybridized with a DIG-labeled probe against U6 snRNA.

### In situ hybridization

Ascidian embryos, larvae, and juveniles at 21, 34, and 42 hpf were collected and fixed in 4% paraformaldehyde (PFA) overnight at 4 °C. DIG-labeled LNA-modified probes (Additional file 5: Table S3) were designed and synthesized by Exiqon (Denmark). Whole-mount in situ hybridization was performed as previously described [37] at a temperature 20 °C below the probe melting temperature. Briefly, after fixation, samples were placed in PBST and washed four times at room temperature. Then, samples were treated with proteinase K at 37 °C for 20 min. After treatment, samples were re-fixed in 4% PFA and washed four times in PBST. After washing, samples were pre-hybridized in a prehybridization buffer for 2 h in a humid chamber. Subsequently, hybridization was conducted at hybridization temperature for 18 h using miRNA LNA probes in a hybridization solution. After hybridization, samples were washed in gradient saline-sodium citrate at the hybridization temperature. Signals of hybridization were detected using alkaline phosphatase-conjugated digoxigenin antibody (Roche) at a 1:2000 dilution. Samples were stained with BCIP/NBT and visualized under a microscope.

### Bioinformatics analysis

Clean data (clean reads) were obtained by removing reads containing poly-N, with 5′adapter contaminants, without 3′ adapter or the insert tag, containing poly A or T or G or C and low-quality reads from raw data. miRDeep2 [38] and miReap (http://sourceforge.net/projects/mireap/) were then utilized to identify miRNAs by mapping clean sRNA reads to predict pre-miRNA structures in the *C. savignyi* genome. Novel and conserved miRNAs were then classified by searching for them against miRBase20.0 (http://mirbase.org/). For miRNA-target prediction, 3′-UTRs of *C. savignyi* mRNAs obtained from the ENSEMBL database were used as potential target sequences. miRNA binding sites were then predicted using miRanda [39] and TargetScan 7.1 (targetscan.org). Targets predicted using miRanda whose expression levels on performing RNA-Seq were negatively correlated with miRNAs were collected for further analysis. The interactions of miRNA and their target genes were visualized as a network using Cytoscape3.4. KOBAS software [40] was utilized to test the statistical enrichment of target gene candidates in KEGG pathways.

For evolutionary analysis, *C. savignyi* miRNAs were compared with miRNAs of 16 other animal species downloaded from miRBase. Only miRNAs supported by experimental evidence were used. The mature miRNAs sequences from different species were clustered according to sequence similarity, to identify conserved miRNA families, requiring alignment ≧ 15 nt which covers the miRNA seed region (position 2-8 nt) and mismatch ≦2. Moreover, at most 1 nt overhang was allowed in the alignment between the 5′ends of mature miRNAs. Then reconstruction of the evolution history of miRNA families were performed using in-house perl scripts, applying the Dollo parsimony criteria.

### Cell culture

Human embryonic kidney 293T cells (HEK293T) were cultured in DMEM (Invitrogen, USA) supplemented with 10% fetal bovine serum (Gibco, USA), penicillin (100 U/ml), and streptomycin (100 μg/ml) in a humidified atmosphere of 5% CO_2_ at 37 °C.

### Luciferase reporter constructs and luciferase assay

Wild-type *C. savignyi* MAPK1–3′-UTR or MAPKK3–3′-UTR fragments containing predicted miRNA target sites were amplified by PCR using *C. savignyi* embryo cDNA as templates. A mutant 3′-UTR construct (pmirGLO-MAPK1–3′-UTR mutant) with a mutation of three nucleotides (Fig. 7a) from the site of MAPK1 or pmirGLO-MAPKK3–3′-UTR mutant with a mutation of seven nucleotides (Additional file 4: Figure S2A) from the site of the MAPKK3 gene were generated by overlap extension PCR. Primer sequences are shown in Additional file 6: Table S4. Both wild-type and mutant 3′-UTRs were cloned into the firefly luciferase coding region of the pmirGLO plasmid (Promega, USA).

For reporter assays, cell transfections were performed using Lipofectamine 3000 (Invitrogen, USA) following the manufacturer’s instructions. One day before transfection, HEK293 cells were passaged in 24-well plates at 70–80% confluence. Then, 0.4 μg of miRNA 3′-UTR plasmid, 1 μl of P3000 regent, and 100 nM miRNA mimics (Genepharma, China) were diluted in 25 μl of medium and 1.5 μl of Lipofectamine 3000 was diluted in 25 μl of medium. Then, the diluted construct was mixed with the diluted Lipofectamine 3000. The mixture was incubated for 5 min at room temperature and was then added to each well containing 500 μl of cells and the medium. Forty-eight hours after transfection, firefly and *Renilla* luciferase activities were measured using the Dual-Luciferase reporter assay system (Promega, USA), and relative reporter activity was normalized to *Renilla* luciferase activity. Each assay was performed in triplicate.

## Conclusions

We identified 165 miRNAs, including 59 novel ones, from the embryos and larvae of *C. savignyi*. Twelve of them showed significant changes in expression before and during metamorphosis. In situ hybridization and northern blotting results revealed that three miRNAs are potentially involved in the signaling regulatory network for the migration and differentiation of mesenchymal cells in larval metamorphosis. We also revealed the target genes of identified 12 miRNAs. Our results not only present a list and profile of miRNAs involved in *Ciona* metamorphosis but also provide informative cues to further understand their function in ascidian larval metamorphosis.

## Additional files

Additional file 1: **Table S1.** Sequences of identified known miRNAs in *C. savignyi.* (DOCX 108 kb)

Additional file 2: **Table S2.** Sequences of identified novel miRNAs in *C. savignyi.* (DOCX 103 kb)

Additional file 3: **Figure S1.** Original images of northern blotting presented in Fig. 5. (PDF 1785 kb)

Additional file 4: **Figure S2.** Validation of the interaction between csa-miR-4018a or csa-miR-4018b and *Mapkk3–*3′-UTR. (PDF 841 kb)

Additional file 5: **Table S3.** Sequences of probes used in northern blotting and in situ hybridization. (DOCX 47 kb)

Additional file 6: **Table S4.** Sequence of primers for mutant constructs used in overlap extension PCR. (DOCX 60 kb)

## Abbreviations

EGF: Epithelial growth factor
ERK: Extracellular regulated protein kinase
JNK: C-Jun N-terminal kinase
LNA: Locked nucleic acid
MAPK: Mitogen-activated protein kinase
MKK: Mitogen-activated protein kinase kinase
MKP: Mitogen-activated protein kinase phosphatase
MSCs: Mesenchymal stem cells
PFA: Paraformaldehyde
Rap: Ras-related protein
TGF-β: Transforming growth factor beta
