# Supplementary material for: Identification and characterization of microRNAs involved in ascidian larval metamorphosis
Source: BMC Genomics. 2018 Mar 1;19:168. doi: 10.1186/s12864-018-4566-4 (PMC5831862; doi:10.1186/s12864-018-4566-4)
Supplement: Supplementary file 1 — Table S1. Sequences of identified known miRNAs in C. savignyi. (DOCX 108 kb) [file 12864_2018_4566_MOESM1_ESM.docx]

Table S1. **Sequences of identified known miRNAs in *C. savignyi***

| **Name** | **Sequence (5'-3')** |
| --- | --- |
| csa-let-7b | UGAGGUAGUAGGUUAUGUUGUU |
| csa-let-7a | UGAGGUAGUAGGUUAUAUCAGU |
| csa-let-7c | UGAGGUAGUAGGUUAUGCAGUU |
| csa-let-7d | UGAGGUAGUUGGUUGUAUUGUU |
| csa-let-7f | UGAGGUAGUGGAUUCUGUAGUU |
| csa-miR-1 | UGGAAUGUAAAGAAGUAUGCGU |
| csa-miR-124 | CGUGUUUAUUGUGGACCUUGCC |
| csa-miR-125 | UCCCUGAGACCCUAAAACGUGA |
| csa-miR-126 | UCGUACCGUGAGUAAUAAAGCA |
| csa-miR-132 | UAUGGCUUCUACAUUCCUGUGUGA |
| csa-miR-133 | UUUGGUCCCCUUCAACCAGCUA |
| csa-miR-135 | UAUGGCUUUCUUAUCCUGUGUGA |
| csa-miR-141 | UAACACUGUCUGGUAAAGAUGC |
| csa-miR-1497 | UUGAAGAAUUGCAGGUGGUAGG |
| csa-miR-15 | AAGCAGCACACAAAACUAUCAU |
| csa-miR-1502b | UGAACUUUACCACGAAAAGGUCUGU |
| csa-miR-1502c | UGAACUUUACCAUGAAAAUGCCUGU |
| csa-miR-1502d | UGAACUUUCCCAGGAAUAAGUCU |
| csa-miR-1510 | UCAAUGUUGUUUUACCGAUUCC |
| csa-miR-153 | UUGCAUAGUAAUAAAAGUGAUCAU |
| csa-miR-1692 | UUAGCUCAGUUGGUAGAGCGUU |
| csa-miR-183 | UAUGGCACUAGUAGAAUUCACUG |
| csa-miR-184 | UGGACGGAGAACUGAUAAGGGC |
| csa-miR-196 | UAGGUAGUUUAAAGUUGUGGAG |
| csa-miR-200 | UAAUACUGCCUGGUAAUGAUGAUU |
| csa-miR-216a | UAAUCUCAGCUGGCAAUCUGUGA |
| csa-miR-216b | UAAUCUCUGCAGGCAACUGUGA |
| csa-miR-217 | UACUGCAUUAGGAACUGAUUGG |
| csa-miR-219 | UGAUUGUCCAAACGCAAUACAGG |
| csa-miR-2478 | UCGAAUCCCACUUCUGACACCA |
| csa-miR-281 | UGUCAUGGAGUUGCUCUCUCAU |
| csa-miR-31 | UGGCAAGAUGUUGGCAUAGCUAU |
| csa-miR-34 | AGGCAGUGUAGUUAGCUAGUUGU |
| csa-miR-367 | UAUUGCACAUUGGAAUGGUACUU |
| csa-miR-375 | CUUGUUCGUUCGGCUCGCGUCG |
| csa-miR-4000e | UGAAACUUCUUCGGAACAGGCCGG |
| csa-miR-4000f | UGAAACUUCGCUGGAACAGGCCGG |
| csa-miR-4001 | CGGAACUUUCCAAGGAACAGGCU |
| csa-miR-4006 | UGGAACAUGCAGAUAAGGGCUG |
| csa-miR-4008 | AGGGUUUAUCCUGUCUGCCACA |
| csa-miR-4009 | UAUUGCACAUGUUGUACCAAC |
| csa-miR-4010 | AACGUAUGUCGAGCAAACAUUGC |
| csa-miR-4011 | ACGGUAGCGUUCACUGUAACA |
| csa-miR-4014 | UUACAGAAAGCGUGUGUGUGCUA |
| csa-miR-4017 | UAAGUGCAUGUAUGGUGCUAUU |
| csa-miR-4018a | CGGAACAUGGUUGGAACGGGCUG |
| csa-miR-4018b | AGGAACAUUCUGUGGAACGGGCUGG |
| csa-miR-4029 | AAAGUGCAACUGUGUAAACCU |
| csa-miR-4030 | AAAGUGCAACUUUGAUAGUGU |
| csa-miR-4031 | ACAUGUACCAUCGCUCUGGGAU |
| csa-miR-4033 | UAAGUGCAUUGCGUGCUCCAG |
| csa-miR-4034 | GCACAGUGCUCUAGGAAAGUA |
| csa-miR-4036 | UGUGCAAAGCCCAAUCAUGUCU |
| csa-miR-4037 | ACAAUUGCACUCGUCUGUACCGCC |
| csa-miR-4038 | CAUUGCACUGUUAUUGAUCUAG |
| csa-miR-4039 | UAUAUGAAGGUGUAAGCUAUUC |
| csa-miR-4040 | UCGUCUUUCUGUAUAUGGUUGUU |
| csa-miR-4041 | CAAUAGGUCUUUAAUUGUUGAG |
| csa-miR-4043 | UUGCAUAGAUUGUCCGAACAUU |
| csa-miR-4044 | AGUUGGUAGACUGAUUUCAGU |
| csa-miR-4045 | CCACAAUGAAAGUAGAUGUCCGUU |
| csa-miR-4046 | UCAGUAACACGAAUACGUCCUGGA |
| csa-miR-4047 | UUGUGUAUCUGAGUGUUUCUCU |
| csa-miR-4048 | GCACCUUGGUCACCAAUAUCUGCU |
| csa-miR-4049 | AAGCUGUGGGCUUUGCCUAAAG |
| csa-miR-4050 | UAGGUAAAUCAAAUUUCAGGCUGU |
| csa-miR-4051 | UUCAACCAUCAUGGCACUAUUGGGU |
| csa-miR-4052 | UCACUUGAAACCCAGGACCUGUUG |
| csa-miR-4053 | UAGUACGUCGUUCUCCGGACGG |
| csa-miR-4054 | UAUGAUUGAUGUUUAAUGGCUCU |
| csa-miR-4055 | UAUUCGGAAAUGUAGGGUGGACG |
| csa-miR-4056 | ACUGAUGUAGAACAAGGCAUGCGC |
| csa-miR-4057 | UUUGCUACUUUCACCAAGAUCAG |
| csa-miR-4059 | UAGCAGCAAAGACAGUAUUGGA |
| csa-miR-4060 | AUUUCACUCUUUCAACUGUUGG |
| csa-miR-4061 | AACACUCUGGCGUUCCGUCAUU |
| csa-miR-4063 | AACACCUUAAUCACCAAUGUGAUUG |
| csa-miR-4064 | AGUCAGGAUAACGGUUAGUUUU |
| csa-miR-4065 | UAUCGACAAGAUCUCUCAGUGGG |
| csa-miR-4066 | ACAGAUGUGGUGCAGGCAUGCA |
| csa-miR-4067 | CAAGGAACGGAAAUGAGCCGUUGG |
| csa-miR-4068 | UAUGCUGAUGAACAAAGGCUUGU |
| csa-miR-4069 | CUACCCUGUAUUUACGCUGUGU |
| csa-miR-4070 | UCGAAUGUUGUGCAGGCACGUGCA |
| csa-miR-4071 | CUUGCUCUUACUGUGACAUCCCU |
| csa-miR-4072 | UUUGUUUUAAGGCUUCAUUUUCU |
| csa-miR-4073 | CAGGAUGUUCGACUUGUUGUGG |
| csa-miR-4074 | UGUUGACGGCGGGGAUGUUAGU |
| csa-miR-4076 | CAACGAGUCCCUGAAAAAUCCCG |
| csa-miR-4078 | UUCACUUUGCCACUGCAGGUCU |
| csa-miR-4085 | UCGCACGUUGUGCAGGCAUGCA |
| csa-miR-4086 | CAUUUUGAUGGCUACCUCCCAA |
| csa-miR-4091 | CAGCUGUUACCUCACUACCCCA |
| csa-miR-4092 | UAGGUCUGUCUACACAACACCGU |
| csa-miR-4093 | AUGCAACAAAACUGCGGUUAUG |
| csa-miR-4099 | CGGAUUGACAUGGUGUCAGAGA |
| csa-miR-4113 | UCUGGUUUUCACUGUGUAAAUG |
| csa-miR-4175 | GGGGGUGUAGCUCAGAUGGUAG |
| csa-miR-4220 | UAGUGCAAUUUGUUGUAGCUUG |
| csa-miR-5001 | AGUACCAUCACGCGCCAUGCC |
| csa-miR-5601 | AUGUGCUUUUGUCUAACCAUGUU |
| csa-miR-5978 | ACCGAGACUAGAGUCACAUGGU |
| csa-miR-7 | UGGAAGACUAGUGAUUUUGUUGUU |
| csa-miR-92a | UAUUGCACUUCCCUAGACUGGU |
| csa-miR-92b | UAUUGCACUUGUCCCGGUCUU |
| csa-miR-92c | UAUUGCACCUGUCCCGGCCGAU |
| csa-miR-96 | UUUGGCACUUGCACAAAAUUGA |
