# Supplementary material for: Identification and characterization of microRNAs involved in ascidian larval metamorphosis
Source: BMC Genomics. 2018 Mar 1;19:168. doi: 10.1186/s12864-018-4566-4 (PMC5831862; doi:10.1186/s12864-018-4566-4)
Supplement: Supplementary file 2 — Table S2. Sequences of identified novel miRNAs in C. savignyi. (DOCX 103 kb) [file 12864_2018_4566_MOESM2_ESM.docx]

Table S2. **Sequences of identified novel miRNAs in *C. savignyi***

| **Name** | **Sequence (5'-3')** |
| --- | --- |
| csa-miR-n1 | UGGAACUUCCUUGAACAGGCUG |
| csa-miR-n10 | ACGACAGUCGUUGAAACACGCC |
| csa-miR-n11 | UUGGCAUGGUCUGUGUCCGGAGUG |
| csa-miR-n12 | UUAUUGCUUCUGAUUGCGCCC |
| csa-miR-n13 | UGAAACUUUCUAGGAACGGGCCU |
| csa-miR-n14 | UGUGAUCUUUGCCGUGUAAAUGAAG |
| csa-miR-n15 | UAUUGCACACGUAUGUACCCU |
| csa-miR-n16 | CGCGUUUGCUGUGGACCUUCUU |
| csa-miR-n17 | AAGUUGGACGCAUUAGGGCUGG |
| csa-miR-n18 | UGAGUUGAGCUCCGAAAAGUGCGA |
| csa-miR-n19 | UGCGUGAUCUUCUGACAACAGUGG |
| csa-miR-n2 | UCGUGUCGCGGUCCGGGACU |
| csa-miR-n20 | ACGGUGGCCAUGGAAGUC |
| csa-miR-n21 | UCUUUGGUGAACUAGUUUUUUG |
| csa-miR-n22 | UGCUGUGCUGUUCAAUUGUGACA |
| csa-miR-n23 | UAAAAAUGCGAGUAGUGAUAGCCU |
| csa-miR-n24 | AUGGGUGACCGCUUGGGAAU |
| csa-miR-n25 | GUUCGAUUCCCGGCCAAU |
| csa-miR-n26 | ACAGACUUGUAGAUAUCGUACA |
| csa-miR-n27 | UAGUUCAACUCCAAUUAGAAUU |
| csa-miR-n28 | UGUCUCUUGCCAUCUCUGUUC |
| csa-miR-n29 | AUGGCGUGGGUUCGAAUCA |
| csa-miR-n3 | UGGAACAUCUAUGUAAGGGCUG |
| csa-miR-n30 | UAGUUGAACUUUAACCGGUAGCGG |
| csa-miR-n31 | UCGGAUAACUUGUGAGGGAAAUC |
| csa-miR-n32 | GUUUUAAUUAUCACUGUUGACC |
| csa-miR-n33 | CGCGUUUGCUGCGGACCUUCUU |
| csa-miR-n34 | CUCGACCUCGAGAGGCGUAGU |
| csa-miR-n35 | CUGGUUUCUGUGUGCCGCUACC |
| csa-miR-n36 | AGUACCAUGGUUGCACUUGUA |
| csa-miR-n37 | UAACGUGGACUUGGAUUUGAUAGC |
| csa-miR-n38 | UGUAGAUUGUACCUGGGGUGU |
| csa-miR-n39 | AAUUCUGGACGAGCGCCGACAA |
| csa-miR-n4 | GGUUCGAUUCCCGGUCUGGGAAC |
| csa-miR-n40 | AUUACUACAGGAUCGCUUGGA |
| csa-miR-n41 | GAUCUUGAUGUAUUGGAUGAUGCUG |
| csa-miR-n42 | AGUGUAAUGUCUAUGGAAGCAGU |
| csa-miR-n43 | AAGAGGAACAGACGGGGGG |
| csa-miR-n44 | ACAGACUUGUAGAUAGCGCACA |
| csa-miR-n45 | UUUCACCUCAAUGAGUAUCAUGUAG |
| csa-miR-n46 | UACAGAAUCGGCACAAGGGUAGAUU |
| csa-miR-n47 | AUUAGAUUGUCACAAAGUGGUUCGC |
| csa-miR-n48 | CGGAACAUGCUGUAUGGCG |
| csa-miR-n49 | GGUUAGCUUUUUGAACUGGAUAGAC |
| csa-miR-n5 | AGGAACAUGCUUGUAAGGGCUG |
| csa-miR-n50 | UUAUUCGGACGGAAUUAAUACU |
| csa-miR-n51 | UGUUCAAGAACUGCAGCAGGAUGUA |
| csa-miR-n52 | CAUUUUGAGGUGGAAUCUGGUC |
| csa-miR-n53 | UUGUCACUACAGAAGCUCGGU |
| csa-miR-n54 | UUGUUGUUCAUAGGUUGCCUUA |
| csa-miR-n55 | AGUUGAACUUUAACCGGUAGCGG |
| csa-miR-n56 | UGGAACGUGUAGUGUUGGCAGAGG |
| csa-miR-n57 | UUUUCCCGUUAGGUUAUGUGU |
| csa-miR-n58 | AAGACGAACGACUGCGAAAGC |
| csa-miR-n59 | UCUCGGUAAUAUACACUCUUGGU |
| csa-miR-n6 | CUACCCUGUAUUUACGCUGUGU |
| csa-miR-n7 | UGAAACUUAUUGGAACAGGCCUU |
| csa-miR-n8 | GCGUGGCCGUGGAAAGAGC |
| csa-miR-n9 | AGAUAGGUCGAUUUCAUCGUGG |
