# Supplementary material for: Identification and characterization of microRNAs involved in ascidian larval metamorphosis
Source: BMC Genomics. 2018 Mar 1;19:168. doi: 10.1186/s12864-018-4566-4 (PMC5831862; doi:10.1186/s12864-018-4566-4)
Supplement: Supplementary file 3 — Figure S1. Original images of northern blotting presented in Fig. 5. (PDF 1785 kb) [file 12864_2018_4566_MOESM3_ESM.pdf]

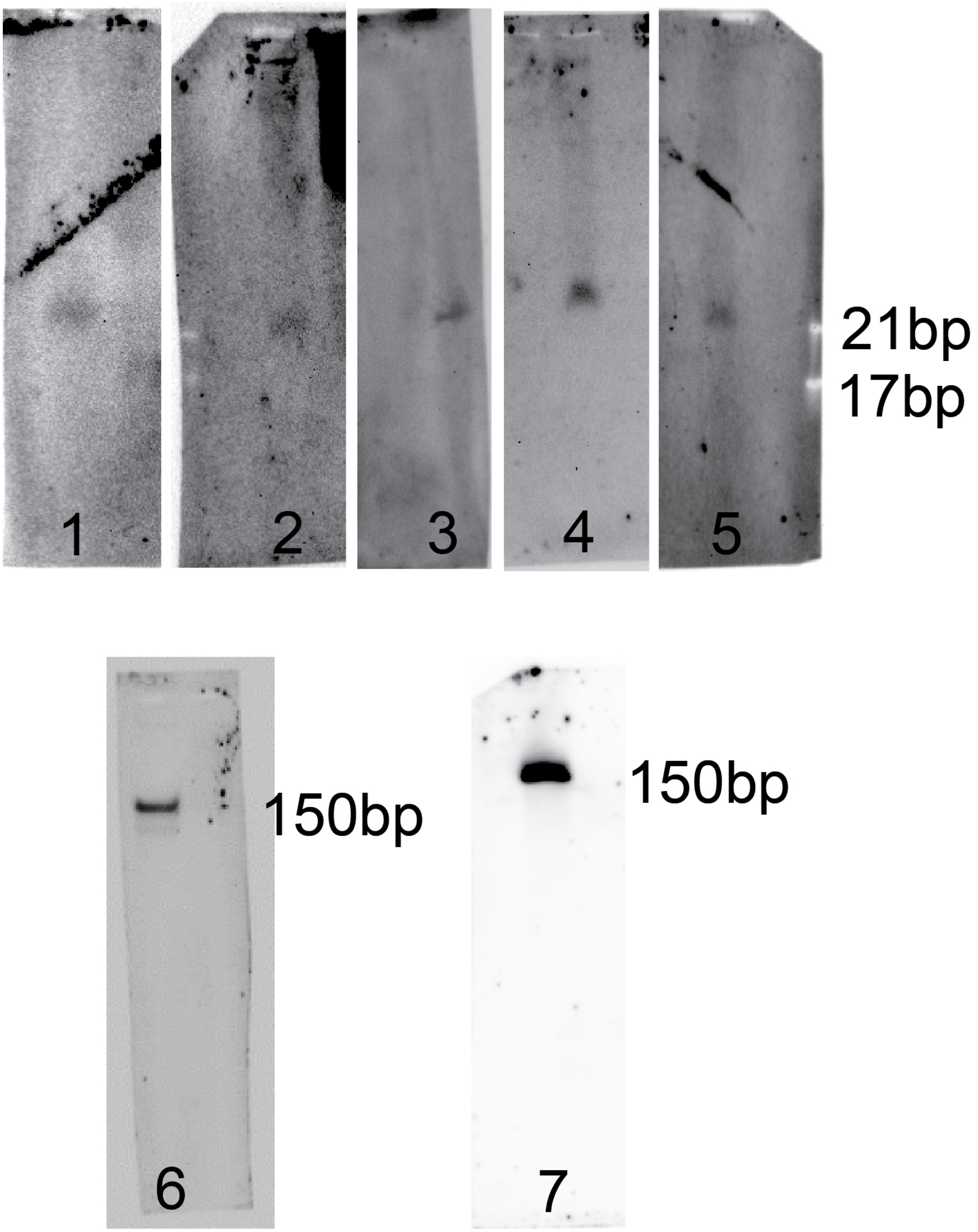

Figure S1. Original images of northern blotting used in Figure 5.

Lane1: csa-miR-4018a; Lane2: csa-miR-4018b; Lane3: csa-miR-4000f; Lane4: csa-miR-4040; Lane5: csa-miR-4086. The U6 snRNA served as the loading control. Lane6: U6 using total RNA extracted from adult; Lane7: U6 using total RNA extracted from embryos.
