# Supplementary material for: Identification and characterization of microRNAs involved in ascidian larval metamorphosis
Source: BMC Genomics. 2018 Mar 1;19:168. doi: 10.1186/s12864-018-4566-4 (PMC5831862; doi:10.1186/s12864-018-4566-4)
Supplement: Supplementary file 4 — Figure S2. Validation of the interaction between csa-miR-4018a or csa-miR-4018b and Mapkk3–3′-UTR. (PDF 841 kb) [file 12864_2018_4566_MOESM4_ESM.pdf]

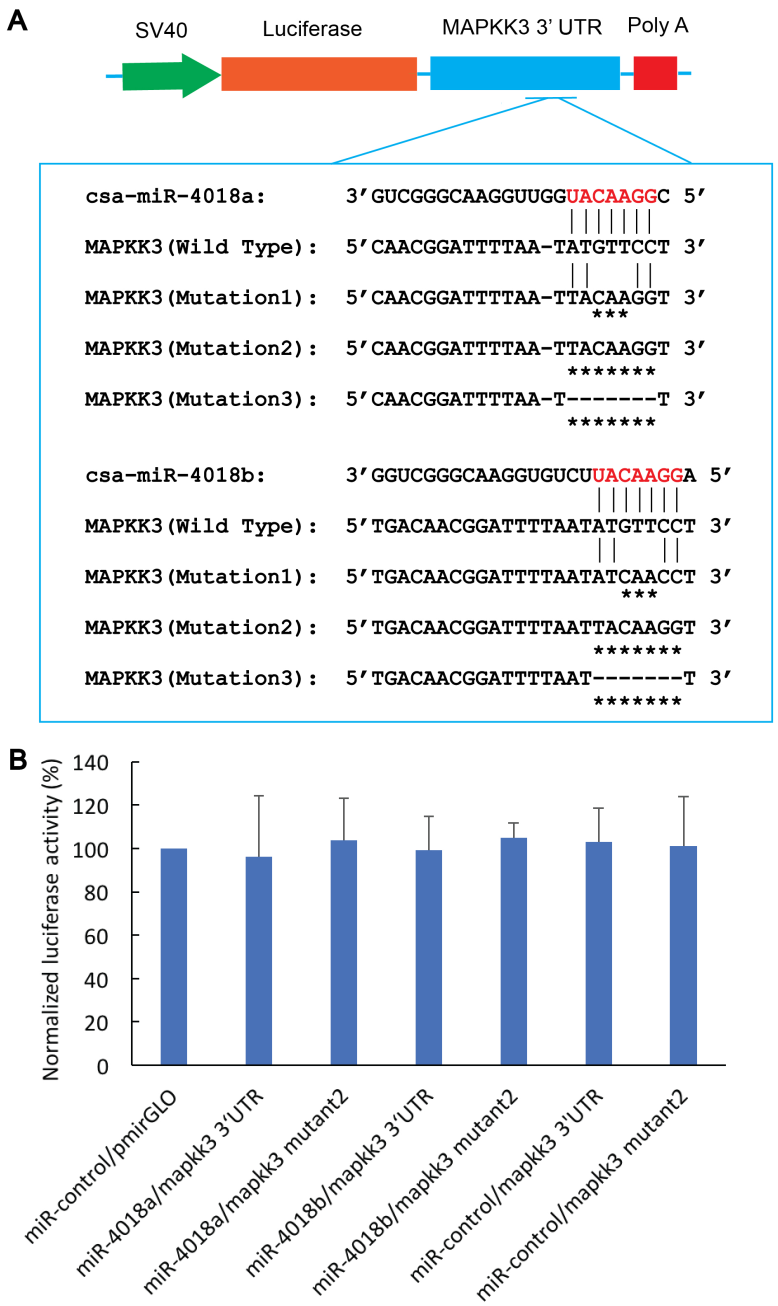

Figure S2. Validation of interaction between miR-4018a, or miR-4018b and *Mapkk3* 3′UTR. (**A**) Schema of the luciferase reporter constructs carrying of target genes 3′UTR used in the luciferase assays. Sequence alignments of miR-4018a or miR-4018b and their predicted target gene *Mapkk3* 3′ UTR or *Mapkk3* 3′UTR mutants were displayed in the blue box. Asterisks under letters indicate the mutant nucleotide in the *Mapkk3* 3′UTR mutant constructs. (**B**) Relative luciferase activity in HEK293T cells co-transfected with pmirGLO-*Mapkk3* 3′UTR or pmirGLO-*Mapkk3*-3′UTR mutant, or the empty vector pmirGLO and either csa-miR-4018a/b mimics, or miR-control. The firefly luciferase values were normalized for transfection with *Renilla* luciferase activity.
