# Supplementary material for: Identification and characterization of microRNAs involved in ascidian larval metamorphosis
Source: BMC Genomics. 2018 Mar 1;19:168. doi: 10.1186/s12864-018-4566-4 (PMC5831862; doi:10.1186/s12864-018-4566-4)
Supplement: Supplementary file 5 — Table S3. Sequences of probes used in northern blotting and in situ hybridization. (DOCX 47 kb) [file 12864_2018_4566_MOESM5_ESM.docx]

Table S3. Sequences of probes used in northern blotting and *in situ* hybridization

| **Probe name** | **Sequence (5’-3’)** |
| --- | --- |
| csa-miR-4040 | /5DigN/AACAACCATATACAGAAAGACGA |
| csa-miR-4086 | /5DigN/TTGGGAGGTAGCCATCAAAATG |
| csa-miR-4055 | /5DigN/CGTCCACCCTACATTTCCGAATA |
| csa-miR-4018a | /5DigN/CAGCCCGTTCCAACCATGTTCCG |
| csa-miR-4018b | /5DigN/CCAGCCCGTTCCACAGAATGTTCCT |
| Scramble (control) | /5DigN/GTGTAACACGTCTATACGCCCA |
