# Supplementary material for: Identification and characterization of microRNAs involved in ascidian larval metamorphosis
Source: BMC Genomics. 2018 Mar 1;19:168. doi: 10.1186/s12864-018-4566-4 (PMC5831862; doi:10.1186/s12864-018-4566-4)
Supplement: Supplementary file 6 — Table S4. Sequence of primers for mutant constructs used in overlap extension PCR. (DOCX 60 kb) [file 12864_2018_4566_MOESM6_ESM.docx]

**Table S4. Sequence of primers for mutant constructs used in overlap extension PCR**

| Primer name | Sequence (5’-3’) |
| --- | --- |
| mapk1utr-mut-F1 | CCTGCCGCTTAAAAAAACAATCGGGATGCCTATTCCCGATTCCCG |
| mapk1utr-mut-R1 | CGGGAATAGGCATCCCGATTGTTTTTTTAAGCGGCAGGAATTACA |
| mapk1utr-mut-F2 | CCTGCCGCTTAAAAAAAGTAAGGGGATGCCTATTCCCGATTCCCG |
| mapk1utr-mut-R2 | CGGGAATAGGCATCCCCTTACTTTTTTTAAGCGGCAGGAATTACA |
| mapkk3utr-m7-F1 | CAACGGATTTTAATATCAACCTCGTTGGATTTTACCCACAGTG |
| mapkk3utr-m7-R1 | TAAAATCCAACGAGGTTGATATTAAAATCCGTTGTCATGTATG |
| mapkk3utr-m7-F2 | CAACGGATTTTAATTACAAGGTCGTTGGATTTTACCCACAGTG |
| mapkk3utr-m7-R2 | TAAAATCCAACGACCTTGTAATTAAAATCCGTTGTCATGTATG |
| mapkk3utr-m7-F3 | CAACGGATTTTAAT*******TCGTTGGATTTTACCCACAGTG |
| mapkk3utr-m7-R3 | TAAAATCCAACGA*******ATTAAAATCCGTTGTCATGTATG |
